# Validation of the efficacy of the NUTRISCORE for the nutritional screening of cancer patients in China

**DOI:** 10.1186/s12885-021-09135-2

**Published:** 2022-01-06

**Authors:** Junren Kang, Hailong Li, Xiaodong Shi, Enling Ma, Wei Chen

**Affiliations:** Department of Clinical Nutrition, Peking Union Medical College Hospital, Chinese Academy of Medical Sciences and Peking Union Medical College, #1 Shuai Fu Yuan, Beijing, 100730 Dongcheng District China

**Keywords:** Cancer patients, Malnutrition, Patient-generated subjective global assessment (PG-SGA), NUTRISCORE, Malnutrition screening tool (MST)

## Abstract

**Background:**

Malnutrition is common in cancer patients. The NUTRISCORE is a newly developed cancer-specific nutritional screening tool and was validated by comparison with the Patient-Generated Subjective Global Assessment (PG-SGA) and Malnutrition Screening Tool (MST) in Spain. We aimed to evaluate the performance of the NUTRISCORE, MST, and PG-SGA in estimating the risk of malnutrition in Chinese cancer patients.

**Methods:**

Data from an open parallel and multicenter cross-sectional study in 29 clinical teaching hospitals in 14 Chinese cities were used. Cancer patients were assessed for malnutrition using the PG-SGA, NUTRISCORE, and MST. The sensitivity, specificity, and areas under the receiver operating characteristic curve were estimated for the NUTRISCORE and MST using the PG-SGA as a reference.

**Results:**

A total of 1000 cancer patients were included. The mean age was 55.9 (19 to 92 years), and 47.5% were male. Of these patients, 450 (45.0%) had PG-SGA B and C, 29 (2.9%) had a NUTRISCORE ≥5, and 367 (36.7%) had an MST ≥ 2. Using the PG-SGA as a reference, the sensitivity, specificity, and area under the curve values of the NUTRISCORE were found to be 6.2, 99.8%, and 0.53, respectively. The sensitivity, specificity, and area under the curve values of the MST were 50.9, 74.9%, and 0.63, respectively. The kappa index between the NUTRISCORE and PG-SGA was 0.066, and that between the MST and PG-SGA was 0.262 (*P* < 0.05).

**Conclusions:**

The NUTRISCORE had an extremely low sensitivity in cancer patients in China compared with the MST when the PG-SGA was used as a reference.

## Background

Malnutrition is common in cancer patients [1, 2]. Cancer and its related inflammatory factors could cause anorexia and skeletal muscle depletion. In addition, anticancer therapy may cause impaired intake, weight loss, and malnutrition. Many cancer patients could die from cancer cachexia and malnutrition [3, 4]. Early nutritional diagnosis and treatment can help to intervene or treat tumor-related malnutrition, increase the tolerance of antitumor treatment, control the side effects of antitumor treatment, and improve the quality of life [5–8].

The Patient-Generated Subjective Global Assessment (PG-SGA) is a standard nutritional assessment tool for cancer patients [9–12]. However, several factors, such as cancer type, stage, and anticancer therapy, may cause malnutrition and were not considered in the PG-SGA [13]. Recently, a new nutritional screening tool called the NUTRISCORE was developed specifically for cancer patients and validated by reference to the PG-SGA and Malnutrition Screening Tool (MST) [14]. The NUTRISCORE was a cancer-specific malnutrition assessment tool, while PG-SGA and MST were not design for cancer patients only and they were widely used for cancer patients in clinic practice.

In the multicenter, cross-sectional study conducted in Spain, the NUTRISCORE was found to have a better performance than the MST. The NUTRISCORE had good agreement with PG-SGA (kappa index = 0.88), and less time was needed for screening with the NUTRISCORE than with the PG-SGA [14]. As a fast and cancer-specific nutritional screening tool, the NUTRISCORE has also been validated in another study in Spain [15]. However, whether it can be used to predict the malnutrition risk of cancer patients in China is unclear. Therefore, we performed a multicenter, cross-sectional study to validate the performance of the NUTRISCORE and MST compared with the PG-SGA in estimating the risk of malnutrition in cancer patients in China.

## Methods

### Study design

Data from an open parallel and multicenter cross-sectional study were retrospectively analysed. Cancer patients from thoracic surgery, gastroenterology, and oncology departments were enrolled in this open parallel and multicenter cross-sectional study in 29 clinical teaching hospitals in 14 Chinese cities in 2018 [16]. The study was approved by the Ethics Committee of Peking Union Medical College Hospital (approval No. S-K 013), and all participants provided written informed consent. Cancer patients were assessed for malnutrition using the PG-SGA, NUTRISCORE, and MST.

Inclusion criteria: 1) diagnosed with an oncologic disease, 2) age over 18 years, 3) signed informed consent; 4) willing and able to complete the questionnaires. Exclusion criteria: 1) incomplete data for calculating for the PG-SGA, NUTRISCORE, and MST.

The primary objective was to evaluate the performance of the NUTRISCORE, MST, and PG-SGA in estimating the risk of malnutrition in Chinese cancer patients. The sensitivity, specificity, positive predictive values, negative predictive values, positive likelihood ratio, negative likelihood ratio, and areas under the receiver operating characteristic (ROC) curve were estimated for the NUTRISCORE and MST using the PG-SGA as a reference.

### Nutritional screening tools

In this study, the NUTRISCORE, MST, and PG-SGA were used to assess and compare the nutritional status of cancer patients and to clarify the applicability of the NUTRISCORE in the nutritional status of Chinese cancer patients.

The NUTRISCORE was used to screen the nutritional status of cancer patients and validated in the Spanish population. It consists of four parts: involuntary weight loss in the last 3 months and poor eating in the last week due to decreased appetite, tumor location/neoplasm, and oncology treatment. Patients who scored ≥5 points were classified as at risk.

PG-SGA is widely used for cancer patients, developed for hospitalized patients, and recommended by the Oncology Nutrition Dietetic Practice Group of the American Dietetic Association [9]. The PG-SGA consists of patients’ self-reported section, food intake, symptoms, activities and function, weight loss and medical section, disease-related nutrition state, metabolic demand, and physical examination. The PG-SGA results were classified as well-nourished (A), moderately malnourished (B), or severely malnourished (C). For the purpose of comparison and consistency with the NUTRISCORE study [14], PG-SGA stages B and C were also classified as a nutritional risk in this study.

The MST has been widely validated in cancer patients, although it was designed for adult acute hospital patients. The MST had only two questions: Have you lost weight recently without trying? Have you been eating poorly because of a decreased appetite? Patients who scored ≥2 points were classified as at risk.

### Data collection

The NUTRISCORE, MST, and PG-SGA scores of cancer patients were calculated by a trained dietician using data from an open parallel and multicenter cross-sectional study. Data were abstracted and inputted independently by two trained investigators to ensure consistency and integrity.

### Statistical analysis

In the NUTRISCORE study [14], the risk for malnutrition in cancer patients was 28.2% for the MST and 22.6% for the NUTRISCORE. It was calculated that approximately 459 participants would provide 95% power to detect a significant difference of 5% (two-sided a = 0.05, β = 0.1).

Measurement data were expressed as the mean ± standard deviation, and data were counted by percentage description. To determine the accuracy of the NUTRISCORE, MST, and PG-SGA and to predict malnutrition in cancer patients, the areas under the receiver operating characteristic curve (AUC) were calculated using the PG-SGA as a reference method. The sensitivity, specificity and kappa index were also estimated. All statistical tests were two-sided, and *P* values < 0.05 were considered statistically significant. Statistical analysis was performed with SPSS software (Version 19, SPSS Inc., IBM, NY, USA).

## Results

A total of 1000 cancer patients were included. The mean age was 55.9 ± 11.8 (range, 19 to 92 years), and 47.5% (*n* = 475) were male. The proportions of cancer patients who received a college education, secondary education, and primary school education were 21.3, 57.7, and 21.0%, respectively. Furthermore, 6.1% of cancer patients had a family cancer history.

All of the pathological diagnoses of cancer patients were officially collected from the medical records. Lung cancer, breast cancer, and leukemia were the most common diseases, accounting for 34.4, 19.6, and 13.1%, respectively (Table 1). Twenty-nine patients (2.9%) had a NUTRISCORE ≥5, 367 patients (36.7%) had an MST ≥ 2, and 450 patients (45.0%) had PG-SGA B and C (Table 1).

**Table 1:** Patient characteristics

Characteristics	n (%)
**Age, y, mean ± SD**	55.9 + 11.8
Range	19–92
**Sex**	
Male	475 (47.5)
Female	525 (52.5)
**Education**	
Primary school	210 (21.0)
Secondary Education	577 (57.7)
College education	213 (21.3)
**Diagnose**	
Lung cancer	344 (34.4)
Breast cancer	196 (19.6)
Gastric cancer	78 (7.8)
Liver cancer	39 (3.9)
Esophageal cancer	28 (2.8)
Colorectal cancer	83 (8.3)
Cervical cancer	24 (2.4)
Ovarian cancer	28 (2.8)
Leukemia	131 (13.1)
Lymphoma	39 (3.9)
Others	10 (0.1)
**Family tumor history**	60 (6.1)
**NUTRISCORE ≥ 5**	29 (2.9)
**MST ≥ 2**	367 (36.7)
**PG-SGA B + C**	450 (45.0)

*BMI* Body Mass Index, *MST* Malnutrition Screening Tool, *PG-SGA* Patient-Generated Subjective Global Assessment

**Fig. 1:**
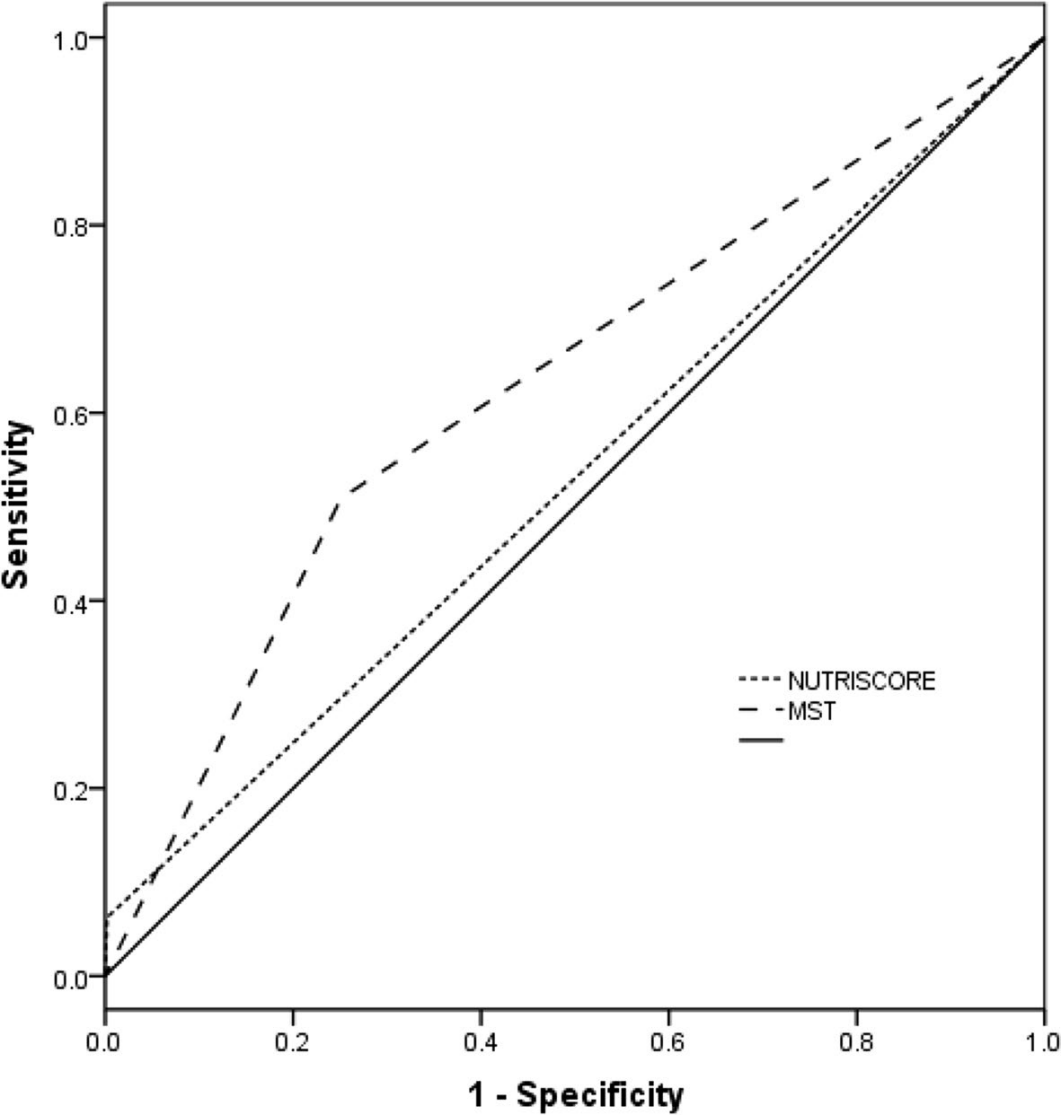
Receiver operating characteristic curves of the NUTRISCORE, MST and PG-SGA

With PG-SGA as a reference method, the sensitivity, specificity, positive predictive values, negative predictive values, and AUC of the NUTRISCORE were 6.2, 99.8, 96.6, 56.5%, and 0.53, respectively. The sensitivity, specificity, positive predictive values, negative predictive values, and AUC of the MST were 50.9, 74.9, 62.4, 65.1% and 0.63, respectively (Table 2). We also compared the AUC for malnutrition in different cancer groups. With PG-SGA as a reference method, the AUC of NUTRISCORE and MST were 0.53 and 0.68 respectively for malnutrition in 344 lung cancer patients. The AUC of NUTRISCORE and MST were 0.50 and 0.58 respectively for malnutrition in 196 breast cancer patients. The sensitivity, negative predictive value, and AUC of the MST were higher than those of the NUTRISCORE, while the NUTRISCORE had higher specificity and positive predictive values (Fig. 1). The kappa index between the NUTRISCORE and PG-SGA was 0.066, and that between the MST and PG-SGA was 0.262 (*P* < 0.05).

**Table 2:** Performance comparison

	NUTRISCORE	MST
Sensitivity % (95% CI)	6.2 (4.2–8.9)	50.9 (46.2–55.9)
Specificity % (95% CI)	99.8 (98.8–100)	74.9 (71.0–78.4)
Positive predictive value % (95% CI)	96.6 (80.4–99.8)	62.4 (57.2–67.3)
Negative predictive value % (95% CI)	56.5 (53.4–59.7)	65.1 (61.2–68.8)
Area under the ROC curve	0.53 (0.49–0.57)	0.63 (0.59–0.66)
Kappa	0.066*	0.262*

*MST* Malnutrition Screening Tool, *ROC* receiver operating characteristic. *P < 0.05

## Discussion

In this study, the NUTRISCORE was first validated in cancer patients in China. Among 1000 cancer patients, 2.9% had a NUTRISCORE ≥5, 36.7% had an MST ≥ 2, and 45.0% had PG-SGA B and C. The NUTRISCORE had higher specificity, while the sensitivity and AUC of the MST were higher when using the PG-SGA as a reference method. The MST had a higher kappa index.

Globally, the incidence and mortality of cancer patients have significantly increased. In general, cancer patients have a hypermetabolic state, increased energy consumption, skeletal muscle depletion, and weight loss [17–19]. The oropharynx malignant tumors could decrease the ability to chew or swallow [20] and the gastrointestinal cancer could cause mechanical obstruction [21, 22]. The chemotherapy lead to weight loss, and weakness [23, 24] and radiotherapy of the head and neck could cause dental caries, stomatitis, and difficulty swallowing [25, 26]. Therefore, malnutrition in cancer patients is common and can lead to increased complications and mortality and prolonged hospital stays [3, 4, 27].

Early diagnosis of malnutrition in cancer patients is particularly important [8]. Higher sensitivity and easy-to-use nutritional screening tools for cancer patients are required to improve clinical outcomes. The PG-SGA is a standard nutritional assessment tool for cancer patients [28]. However, several factors, such as cancer type, stage, and anticancer therapy, may cause malnutrition and were not considered in the PG-SGA [13]. PG-SGA was not cancer-specific. In fact, the incidence of malnutrition differs across different types of cancers. The incidence of malnutrition in pancreatic cancer, gastrointestinal cancers, esophageal cancers, and hematopoietic stem cell transplantation is higher, while the incidence of malnutrition in breast cancer or prostate cancer is lower [29]. Metastatic cancers or advanced cancers were more likely to develop malnutrition [29, 30]. Anticancer chemoradiotherapy may also lead to malnutrition [31]. Therefore, cancer-specific nutritional screening tools may be needed.

As a fast and cancer-specific nutritional screening tool, the NUTRISCORE was developed and validated in the Spanish population. The NUTRISCORE not only contains weight loss and decreased appetite but also includes cancer type, location, and anticancer treatment. To validate the performance of the NUTRISCORE in cancer patients in China, we enrolled 1000 cancer patients and found that only 2.9% of them had a NUTRISCORE ≥5, while the proportion was 22.6% (*N* = 394) in the study from Spain. In addition, the sensitivity, AUC, and kappa index of the NUTRISCORE were lower than those of the MST using the PG-SGA as a reference method, while the NUTRISCORE was found to have a better performance than the MST in the Spain study [14].

This difference may be due to the different sample distributions of the two studies. In our study, the top three cancers (67.1%) were lung cancer (34.4%), breast cancer (19.6%), and leukemia (13.1%), while the scores of breast cancer and leukemia were 0 points in the NUTRISCORE. In the NUTRISCORE study, the top three cancers (45.7%) were abdominal and pelvic cancer (liver, biliary tract, renal and gynecologic cancer, 18.8%), breast cancer (14.5%) and head and neck cancer (12.4%). Malnutrition is more common in head and neck cancer [13, 29]. The distribution of the study sample may cause bias. Second, some of the patients in this study were inpatients, while the NUTRISCORE was designed for outpatient patients.

In our study, the NUTRISCORE had higher specificity and positive predictive values than the MST when using the PG-SGA as a reference method, which was consistent with a study conducted in Spain [14]. As a cancer-specific nutritional screening tool, the NUTRISCORE was associated with good specificity.

However, several cancer-specific factors, such as metastasis, tumor staging, and the number of courses of chemotherapy, are not included in the NUTRISCORE. Metastasis is related to malnutrition and clinical outcomes [30]. Solid tumors and hematological malignancies have different staging systems. Malnutrition related to systemic inflammation [32] was also not included in the NUTRISCORE. Whether a single cancer-specific nutritional screening tool is more specific should be discussed. For example, Onodera’s prognostic nutritional index has been used for evaluating malnutrition in gastrointestinal cancer patients [33–35]. In addition, in our study, the cancer-specific NUTRISCORE did not show better performance than the MST, which only included weight loss and decreased appetite. Cancer-specific factors were also not included in the diagnostic criteria of cancer cachexia [32, 36]. In view of this, standard nutritional screening tools such as the MST and NRS2002 [37] may be suitable for cancer patients. The NRS2002 was developed for hospitalized patients and recommended by the European Society for Clinical Nutrition and Metabolism (ESPEN) [38]. It consists of three parts: severity of disease, impaired nutritional status and age. ≥3 points was classified as nutritional risk [37]. The prognostic ability of the NRS2002 in cancer patients was validated in a new study published in Ann Oncol in 2021 [39]. The prognostic ability of both the NUTRISCORE and NRS2002 will be validated in our future work.

This study had some limitations. First, the distribution of cancer patients may strongly influence the results of the study. There were more breast cancer patients in this study (19.6% vs. 14.5%), and these patients were usually not malnourished. The groups of cancer patients between the current study and NUTRISCORE study should be more comparable in theory. However, considering that the NUTRISCORE was developed as a cancer-specific nutritional screening tool for all cancer patients, not only for the cancer patients in the NUTRISCORE study, the results still hold. In addition, the study was performed with data from an open parallel and multicenter cross-sectional study, which was conducted in a nonselected population of cancer patients. The distribution of cancer types might be closer to the true circumstances of cancer patients in China [40–42]. Second, most of the patients in this study were inpatients. Third, the NUTRISCORE, MST, and PG-SGA scores of cancer patients were calculated from the database, which may cause bias. Fourth, the genetic background of subjects was likely to be related to nutritional status [43]. However, the family tumor history was not discussed and excluded in the NUTRISCORE study [14] and its validated study in Spain [15]. Family tumor history was not included in the NUTRISCORE, MST, PG-SGA or NRS2002. The genetic background of cancer subjects will be discussed in our future basic and clinical research. Further large sample studies are needed.

## Conclusions

The NUTRISCORE had an extremely low sensitivity in cancer patients in China compared with the MST when using the PG-SGA as a reference. Further studies are needed.

## Data Availability

The datasets used during the current study are available from the corresponding author upon reasonable request.

## Abbreviations

PG-SGA: Patient-Generated Subjective Global Assessment
MST: Malnutrition Screening Tool
BMI: Body Mass Index
ROC: Receiver operating characteristic
